# Compartmentalization of Host Multi-Omics Responses in Lower Respiratory Tract Infection: Discordance Between Airway-Local and Systemic Molecular Profiles—A Narrative Review

**DOI:** 10.3390/diagnostics16213489

**Published:** 2026-10-25

**Authors:** Shunjun Tang, Aiyi Hao, Bin Su, Bin Liu

**Affiliations:** Integrative Medical Center, Tianjin University/Characteristic Medical Center of Chinese People's Armed Police Force, Tianjin 300162, China; tsj0916yx@163.com (S.T.); haoaymails@163.com (A.H.)

**Keywords:** lower respiratory tract infection, community-acquired pneumonia, multi-omics, bronchoalveolar lavage fluid, biomarkers, compartmentalization, cross-compartment profiling

## Abstract

Lower respiratory tract infection (LRTI) remains a leading cause of infection-related mortality. Peripheral blood multi-omics shows promise for pathogen discrimination and severity prediction, yet how well blood reflects airway-local responses in LRTI other than coronavirus disease 2019 (COVID-19) is unclear. We searched PubMed, Web of Science Core Collection, and Europe PMC (January 2021 to August 2026); after de-duplication and dual screening, 29 of 817 unique records were included (Layer A: ≥2 host functional omics layers, *n* = 23; Layer B: single omics platform with paired airway–blood specimens, *n* = 6), plus four COVID-19 benchmarks. Blood-based multi-omics distinguished bacterial, viral, and *Mycoplasma pneumoniae* (*M. pneumoniae*) LRTI; severity models performed promisingly within their discovery cohorts. In the two studies reporting airway-versus-blood protein inventories, the airway showed more differentially expressed proteins (DEPs) than blood (airway-to-blood DEP count ratios ≈ 3.6–9.3; descriptive study-specific measures), and cross-compartment correlations were predominantly weak (r ≈ −0.3 to +0.3) in the only paired viral LRTI study; interferon-stimulated gene 15 (ISG15; r = 0.79) was a notable exception. Paired evidence derives mostly from a pediatric cohort, hospital-acquired pneumonia, and acute respiratory distress syndrome (ARDS); adult community-acquired LRTI remains under-represented. Current evidence suggests that peripheral blood provides an incomplete, context-dependent representation of airway-local responses; closing this gap requires adult longitudinal paired cohorts, non-invasive airway sampling, improved integration algorithms, and multimodal data fusion.

## 1. Introduction

Lower respiratory tract infection (LRTI) accounts for approximately 2.2 to 2.5 million deaths annually [1]. Among adults hospitalized with community-acquired pneumonia (CAP), roughly 17–21% progress to severe disease [2], with mortality rates ranging from 18.9% in a Chinese multicentre severe CAP cohort to nearly 50% in intensive care unit (ICU) settings [2,3]. LRTI exhibits marked heterogeneity in etiology, clinical presentation, and host immune response: different pathogens can produce overlapping clinical syndromes, while a single pathogen can cause outcomes ranging from self-limiting mild illness to fatal acute respiratory distress syndrome (ARDS), depending on host factors [4,5].

Conventional microbiological diagnostics, such as culture and antigen testing, are often slow and lack sensitivity. While metagenomic next-generation sequencing (mNGS) has broadened the scope of pathogen detection, it does not capture the host’s immune response state [6]. Concurrently, routine inflammatory markers (e.g., procalcitonin and C-reactive protein) demonstrate limited capacity to discriminate between pathogen classes [7,8,9]. These technologies primarily address pathogen identification rather than characterizing the host’s pathophysiological state.

The coronavirus disease 2019 (COVID-19) pandemic drove large-scale investments in host multi-omics research, establishing methodological benchmarks for analyzing host responses across different biological tiers during acute infection. For instance, Su et al. integrated peripheral blood mononuclear cell (PBMC) transcriptomics with plasma proteomics and metabolomics, revealing a sharp molecular-state shift between mild and moderate disease [10]. Lee et al. and Stephenson et al. utilized single-cell multi-omics to delineate the metabolic differentiation and functional exhaustion of immune cells along a severity gradient [11,12]. These milestone studies, together with complementary single-cell atlases of airway-resident immune cells in COVID-19 [13,14], established a paradigm of profiling host responses across distinct biological compartments (airway and blood). However, these methodological advances have not been adequately transferred to the non-COVID LRTI domain.

An important caveat frames the evidence discussed in this review. The available studies span biologically and clinically distinct conditions—community-acquired pneumonia (CAP), hospital-acquired pneumonia (HAP), viral LRTI, *Mycoplasma pneumoniae* (*M. pneumoniae*) pneumonia, ARDS, and COVID-19—which differ in pathogenesis, host response, and clinical course. The paired airway–blood evidence is concentrated in specific settings: one adult CAP cohort (single-platform proteomics), a HAP cohort, aspiration pneumonia, ARDS, a mixed-aetiology severe pneumonia cohort, and pediatric viral LRTI. We therefore interpret the evidence as a hierarchy: direct adult CAP evidence first, followed by adult LRTI of other aetiologies, pediatric studies, and finally COVID-19 studies, which serve strictly as methodological benchmarks rather than as primary evidence. Throughout the review, findings are attributed to the specific population and pathogen in which they were observed, and conclusions are drawn only to the extent supported by the corresponding tier of evidence.

In clinical practice, peripheral blood is the most accessible specimen, but whether it accurately reflects the airway-local immune microenvironment remains a critical question. Emerging evidence suggests the presence of significant “compartmentalization” in respiratory diseases, wherein molecular signals in local tissues diverge from those in the systemic circulation [15,16]. If peripheral blood multi-omics is to be leveraged for precise diagnosis, severity stratification, or pathogen inference in LRTI, it is imperative to determine the extent to which blood-derived signals represent the pathophysiological processes occurring in the lung.

This review aims to evaluate the current evidence on host functional multi-omics in non-COVID LRTI, with a specific focus on the concordance and discordance between airway and blood signals. We analyze the biological determinants driving this compartmentalization, identify evidence gaps, and outline future research directions. To ensure conceptual clarity, the core terminology used throughout this review is defined in Box 1.

Box 1 **Key Terminology.**

    Host functional multi-omics: The combined application of two or more of the following host molecular layers—transcriptomics (including single-cell, spatial, and non-coding RNA profiling), proteomics, metabolomics, lipidomics, and epigenomics (e.g., DNA methylation, chromatin accessibility)—to characterize host molecular responses during infection.
    Cross-compartment profiling: Parallel molecular characterization of airway-local specimens (e.g., bronchoalveolar lavage fluid [BALF], tracheal aspirate, lung tissue) and peripheral blood collected from the same individual at the same clinical time point. Studies that collect paired specimens but analyse each compartment separately do not provide cross-compartment comparisons.
    Compartmentalization (biological): The observation that molecular signals measured in the airway and peripheral blood exhibit limited overlap, weak correlation, or discordant directionality, reflecting the biological autonomy of local tissue immune responses.
    Inter-compartment correlation: The Pearson or Spearman correlation coefficient calculated for the abundance of the same molecule in paired airway and blood specimens.
    Multi-modal data fusion: The computational integration of multiple omics data types with non-omics clinical data (e.g., microbiology, radiology, physiological scores).

## 2. Materials and Methods

### 2.1. Search Strategy

This review is a narrative review with a systematic identification strategy. We searched three databases—PubMed, Web of Science Core Collection, and Europe PMC—for articles published between 1 January 2021, and 31 August 2026. The search strategy combined free-text terms across three core domains: (1) disease scope (e.g., “community-acquired pneumonia”, “lower respiratory tract infection”, “ARDS”); (2) omics technologies (e.g., “multi-omics”, “proteomics”, “metabolomics”, “single-cell”); (3) specimen types (e.g., “bronchoalveolar lavage”, “plasma”, “blood”). The same conceptual blocks were adapted to each database’s field syntax; the complete search strategies are provided in Table S1. Three of the four Layer C methodological benchmarks were identified through citation indexing rather than through the database search; the fourth (Eddins et al.) was identified by the present search and retained as a benchmark.

### 2.2. Inclusion and Exclusion Criteria: A Layered Strategy

To address the dual objectives of evaluating multi-omics integration and cross-compartment concordance, we prospectively defined a layered inclusion strategy:

Layer A (Core Analysis): Human non-COVID LRTI studies applying ≥2 host functional omics layers. Omics layers were counted by molecular layer rather than by platform (e.g., bulk RNA-seq, single-cell RNA-seq, spatial transcriptomics, and non-coding RNA profiling constitute one transcriptomic layer; CyTOF and plasma proteomics constitute one proteomic layer; DNA methylation and chromatin accessibility constitute one epigenomic layer; lipidomics and metabolomics are counted separately). This threshold was established because the primary objective is to evaluate multi-molecular integration strategies rather than the diagnostic performance of a single omics modality.

Layer B (Supplementary Cross-Compartment Evidence): Non-COVID LRTI studies utilizing a single omics platform but providing paired airway–blood specimens from the same individuals. This layer ensures that critical evidence regarding lung–blood molecular concordance is included.

Layer C (Methodological Benchmarks): Seminal multi-omics studies in COVID-19, listed separately from the primary evidence and therefore not subject to the database search window. These are cited in the Introduction and in Section 4.4 to provide methodological reference points and contribute no data to the evidence synthesis; the four benchmarks are listed in Table S3.

Exclusion criteria comprised: studies restricted solely to the microbiome or pathogen genomics; omics applications used exclusively for pathogen identification; in vitro or animal models; reviews, case reports, editorials, errata, and other non-original research records; and studies that met no layer criterion (e.g., insufficient omics layers without paired specimens).

### 2.3. Study Selection and Data Extraction

After de-duplication of the 1777 retrieved records, 817 unique records remained; 60 records with ineligible publication types were removed before screening. Two investigators independently screened the titles and abstracts of the remaining 757 records; the 73 records on which they disagreed were resolved by a third adjudicator. Potentially eligible full texts were evaluated independently. The selection process followed the Preferred Reporting Items for Systematic Reviews and Meta-Analyses (PRISMA) guidelines (Figure 1). Extracted data included study design, sample size, LRTI subtype, specimen types, omics layers, inter-compartment correlation metrics, key findings, and study limitations (Table 1 and Table S2).

### 2.4. Pre-Specified Limitations

Given the heterogeneity among the included studies regarding omics platforms, specimen preprocessing protocols, and clinical severity grading, a quantitative meta-analysis was deemed unfeasible. Formal risk-of-bias tools (e.g., ROBINS-I) are designed for interventional or diagnostic-accuracy questions and are poorly suited to heterogeneous exploratory omics cohorts; instead, study-quality parameters—design, sample size, comparators, validation strategy, and platform characteristics—were tabulated for every included study (Table 1 and Table S2) and carried into the synthesis. In addition, the three-database search may still have missed studies not indexed in these databases or published in languages other than English.

## 3. Peripheral Blood Multi-Omics in LRTI

Before exploring the cross-compartment relationship, we review multi-omics studies based solely on peripheral blood. The Layer A evidence base comprises 23 studies (Table S2), of which eight representative studies—selected to illustrate the range of study designs, pathogens, and analytical strategies in the evidence base—are discussed in detail, while the remaining studies are cited briefly here and summarised in Table S2. Two of these eight are compartment-resolved studies (Lin et al.; Song et al.) discussed in Section 4. These studies demonstrate that molecular profiles in the systemic circulation can reflect host response differences driven by distinct pathogens and disease severity.

### 3.1. Bacterial Infections: Plasma Signatures in CAP

In adult CAP, bacterial infection triggers specific systemic inflammation and metabolic remodeling. Rischke et al. conducted a combined plasma proteomic, metabolomic, and lipidomic analysis of 58 propensity-score-matched adult CAP patients (from 69 enrolled). They found that CLEC4D (a C-type lectin receptor recognizing bacterial glycolipids) and EN-RAGE/S100A12 were concordantly upregulated in peripheral blood, forming a core protein signature distinguishing bacterial CAP. At the lipidomic level, bacterial CAP was characterized by elevated phosphatidylcholines (PCs) and ether-linked phosphatidylcholines (PC-O) [18]. Furthermore, Saballs et al. used nuclear magnetic resonance (NMR) to profile the sera of adult CAP and COVID-19 patients, revealing that both exhibited significant hypolipidemia, with profound glutamine depletion observed in severe cases—likely reflecting the high metabolic demands of proliferating immune cells [20,32]. Monocyte-focused studies have delineated the cellular basis of these systemic signatures: epigenetic–transcriptomic integration linked immune tolerance to a cholesterol-biosynthesis program in CAP monocytes (Brands et al.) [33], and glycolytic reprogramming with intracellular pyruvate accumulation accompanied blunted cytokine responses, yet intracellular pyruvate levels positively correlated with the IL-1β and IL-10 released by patients’ monocytes, suggesting that pyruvate may partially maintain cytokine production capacity (Otto et al.) [34]. Deep proteomic profiling mapped 1340 differentially abundant monocyte proteins with three metabolic types (Chouchane et al.) [35]. Longitudinal lipidomics resolved monocyte and neutrophil lipid remodelling (Schuurman et al., 2024) [36], and primed neutrophils with inflammatory and glycolytic programs were identified in ward-level CAP (Schuurman et al., 2023) [37]. In severe CAP with acute respiratory failure, metabolomics and lipidomics distinguished COVID-19 from CAP (Ibáñez-Prada et al.) [38].

### 3.2. Viral LRTI: Lipid Remodeling and Immune Exhaustion

Respiratory viral infections leave a molecular imprint in peripheral blood distinct from that of bacterial infections [39,40]. Rischke et al. demonstrated that the plasma of viral CAP patients was enriched with linoleic acid-derived oxidized lipids (EpOME–DiHOME) generated via the CYP450/sEH pathway [18,41]. At the proteomic level, LAG-3, TRAIL, and LAMP3 were coordinately upregulated, forming an immune checkpoint–apoptotic effector module. The upregulation of LAG-3 suggests that viral-induced lipid remodeling may be linked to a state resembling T-cell exhaustion [18,42]. Single-cell profiling has refined these viral signatures: peripheral immune features diverged between COVID-19, influenza CAP, and other-pathogen CAP (Schuurman et al., 2021) [43]. Multi-omics profiling of serum identified and validated diagnostic metabolites in pediatric RSV pneumonia (Huang et al.) [44], and systems immunology resolved disease-specific immune programs between RSV and SARS-CoV-2 in infants (Thibodeau et al.) [45]. Plasma proteomics–metabolomics amplified inflammatory signatures in viral pneumonia complicated by pulmonary aspergillosis (Li et al.) [46].

### 3.3. Mycoplasma pneumoniae: Tryptophan Metabolic Reprogramming

Among atypical pathogens, the multi-omics profile of *Mycoplasma pneumoniae* has been relatively well characterized. Tang et al. performed plasma proteomic and metabolomic profiling in pediatric *M. pneumoniae* pneumonia (MPP), showing that indoleamine 2,3-dioxygenase 1 (IDO1) expression increased in a gradient corresponding to disease severity. This upregulation was accompanied by tryptophan depletion and kynurenine accumulation, leading to a Kyn/Trp ratio imbalance [21]. This “dual-branch metabolic reprogramming” (upregulation of the kynurenine pathway with concomitant downregulation of the gut-derived indole pathway) may drive the immune microenvironment toward a state of tolerance, a phenomenon also observed in sepsis [47,48,49]. Complementing these plasma findings, plasma lipidomics and metabolomics identified a 13-metabolite panel discriminating *M. pneumoniae* bronchitis from pneumonia, with urine also profiled (Chen et al.) [50], while BALF multi-omics in pediatric MPP implicated efferocytosis and the APAF1–SERPINB5–L-arginine axis in severity (Li et al., 2026 [51]; analysed as a BALF-internal finding; see Section 4.2).

### 3.4. Predictive Modeling in CAP and Broad LRTI

When LRTI progresses to systemic inflammation, host multi-omics profiles undergo a fundamental shift, often characterized by lipoprotein depletion and complement consumption [52,53,54]. Multiple studies have applied machine learning algorithms to high-dimensional blood omics data for severity prediction, aetiological discrimination, and prognosis. Wang et al. developed a panel comprising CRP, LBP, and three metabolites in a discovery cohort of 50 pediatric patients (20 with severe CAP) and validated it in an independent cohort of 129 subjects, discriminating severe from non-severe CAP with an AUC of 0.991 [19]. In adult severe CAP, Kuang et al. identified severity-associated markers (S1P↓/APOC1↓/APOA2↓/S100P↑); an integrated proteomic–metabolomic model (121 features) achieved a macro-average AUC of 0.944 for three-class classification (estimated on a held-out 7:3 train/test split without external validation) [22]; meanwhile, Li J et al. revealed concurrent overactive inflammation and suppressed lipid metabolism in adult severe patients [23]. A multi-omics model combining miRNAs, proteins, and metabolites has also been proposed for distinguishing bacterial from non-bacterial pneumonia (Hu et al.) [55], and lower-airway microbiome–metabolome clusters were associated with prognosis in acute severe LRTI (Zhan et al.) [56]. In ARDS, neutrophil transcriptional states were compared between bacterial and COVID-19 ARDS (Sinha et al.) [57]. Notably, Li J et al. pointed out that although adult and pediatric severe CAP share certain inflammatory pathways, the proportional composition of the altered lipid classes differed between the two cohorts, suggesting that age should be considered when constructing biomarker models [23]. Furthermore, these multi-omics models demonstrated promising performance within their respective discovery and validation cohorts; however, validation was limited to internal cross-validation or small independent test sets, and no study has performed a rigorous, prospective “head-to-head” comparison between multi-omics panels and traditional biomarkers in an independent LRTI cohort.

The aforementioned studies indicate that peripheral blood multi-omics holds potential for pathogen discrimination and severity stratification. However, most of these investigations rely exclusively on blood specimens, and BALF-based multi-omics findings remain rare (Section 4.2). This raises a translational question: to what extent do these molecular signatures captured in the systemic circulation reflect the local immune events occurring in the alveolar microenvironment?

## 4. Paired Airway–Blood Multi-Omics: Cross-Compartment Discordance

To determine whether peripheral blood can represent the pulmonary microenvironment, it is essential to rely on parallel multi-omics profiling of paired airway and blood specimens collected simultaneously from the same individuals. In the non-COVID LRTI domain, such studies are rare. Synthesizing the six studies reporting cross-compartment comparisons (encompassing pediatric viral LRTI [26], adult *P. aeruginosa* HAP [24], mixed-aetiology severe pneumonia [28], ARDS [29,31], and adult CAP [30]), a consistent phenomenon emerges: biological compartmentalization (Figure 2).

Before synthesizing this evidence, we state an attribution framework to avoid conflating distinct sources of discordance. An observed discordance between airway and blood molecular profiles can arise from four classes of factors: biological compartmentalization, temporal mismatch, sampling effects, and analytical effects. The quantitative and qualitative divergence described in Section 4.1 and Section 4.2 primarily reflects biological compartmentalization, whereas temporal asynchrony (Section 5.3) and technical factors such as BALF dilution and platform differences (Section 5.4) contribute temporal, sampling, and analytical components; bridging molecules (Section 4.3) illustrate the exceptions. Throughout, we interpret correlations as descriptive evidence of concordance or discordance, not as proof of molecular transport.

### 4.1. Quantitative Asymmetry: Differences in Local vs. Systemic Remodeling

A primary observation from cross-compartment analysis is the disparity in molecular signal magnitude. Lydon et al. performed SomaScan proteomic analysis on paired tracheal aspirates (TAs) and plasma from critically ill children with viral LRTI, identifying 200 differentially expressed proteins (DEPs) in the airway compared to 56 in plasma, an airway-to-blood DEP count ratio of approximately 3.6 [26]. This pattern is also observed in bacterial infections: in an adult *P. aeruginosa* HAP cohort, Lin et al. found that the number of DEPs in BALF (93 proteins) was approximately 9.3-fold higher than the number of key DEPs identified in serum (*n* = 10) [24]. These ratios are descriptive study-specific measures rather than pooled effect sizes, because DEP counts depend on statistical thresholds, sample size, platform, normalization, and specimen characteristics. Within these studies, peripheral blood multi-omics captured only a fraction of the molecular alterations triggered by pulmonary infection.

### 4.2. Qualitative Divergence: Local Retention of Pathogen-Specific Immune Pathways

Beyond quantitative asymmetry, the airway and blood exhibit qualitative divergence in activated immune pathways. Studies demonstrate that key immuno-metabolic axes orchestrating local pulmonary pathology often evade detection in peripheral blood. For instance, multi-omics profiling of BALF from pediatric *M. pneumoniae* pneumonia identified an APAF1–SERPINB5–L-arginine regulatory axis that drives inflammatory responses [51]; arginine metabolism is a shared node in these pathways, as the macrophage metabolism of apoptotic cell-derived arginine sustains continual efferocytosis and injury resolution [58]. Similarly, proteins associated with neutrophil extracellular traps (NETosis), such as LCN2 and CALR, which serve as a core defense mechanism against *P. aeruginosa*, displayed high abundance in the airway but were markedly attenuated in serum [24]. In pediatric influenza-associated ARDS, Song et al. reported spatially compartmentalized repair niches: survivors showed regionally restricted repair with preserved alveolar type II cells and elevated KRT17, whereas the fatal case exhibited diffuse immune activation and pro-fibrotic CTHRC1+ fibroblast programs [25]. In the same cohort, single-cell profiling of BALF traced a local transition from FCN1-high inflammatory macrophages at the acute phase to FABP4+ resident-like macrophages at recovery, alongside an increase in KRT17+ stress-repair epithelial cells; circulating KRT17 was higher in survivors and was proposed as a candidate prognostic biomarker [25]. This may reflect that highly pathogen-specific local immune signals are diluted or lost upon entering the systemic circulation.

### 4.3. Bridging Molecules: Exceptions to Cross-Compartment Weak Correlation

Due to the aforementioned quantitative and qualitative divergence, correlations between airway and blood protein abundances are predominantly weak; in the only paired viral LRTI study (Lydon et al.), Pearson correlation coefficients between tracheal aspirate and plasma proteins ranged mostly from −0.3 to +0.3 [26]. Nevertheless, a subset of molecules can act as “bridging molecules,” maintaining consistency across compartments; in that same study, the interferon-stimulated gene 15 protein (ISG15) was one of the rare proteins exhibiting a strong correlation (r = 0.79) between TA and plasma [26]. Additionally, small-molecule metabolites such as the kynurenine/tryptophan ratio have been proposed as blood-based surrogates of local metabolic reprogramming, based on plasma multi-omics profiling in pediatric *M. pneumoniae* pneumonia [21]; whether such metabolites track concordantly across airway and blood compartments remains to be tested in paired designs. Identifying these molecules is crucial for developing viable blood-based surrogate biomarkers.

### 4.4. The Critical Blind Spot: The Absence of Paired Multi-Omics Data in Adult Community-Acquired LRTI

Although the paired studies discussed above reveal a consistent pattern of cross-compartment discordance, the available evidence remains structurally constrained. Cross-compartment comparisons of two or more host omics layers are currently available only from a single adult hospital-acquired pneumonia cohort (*P. aeruginosa*), whereas the remaining paired studies each profiled a single platform—in pediatric viral LRTI [26], aspiration pneumonia [27], mixed-aetiology severe pneumonia [28], ARDS [29,31], and adult CAP [30].

Adult community-acquired LRTI is not entirely unrepresented. Semenova et al. collected matched BALF and plasma from mechanically ventilated adults with community-acquired pneumonia and, using longitudinal mass-spectrometry proteomics, reported pneumonia type-specific local protein signatures, several of which were also detectable in plasma [30]. Notably, immunoglobulin V-segments enriched in the airway were more pneumonia type-specific than those detected systemically, consistent with the view that pathogen-specific immune signals are most sharply resolved within the lung. This study, however, profiled a single omics platform and therefore cannot address how distinct molecular layers are integrated across compartments. Matched airway–blood multi-omics has, however, been implemented in COVID-19 (Eddins et al. [17]); no corresponding design is yet available for non-COVID-19 adult CAP.

To our knowledge, no published study has applied two or more host omics layers to paired airway–blood specimens from adult community-acquired LRTI (CAP). Given the differences between adults and children in immune maturity, comorbidity burden, and pathogen distribution, directly extrapolating pediatric cross-compartment findings to adult CAP requires dedicated validation.

## 5. Mechanisms Driving Compartmentalization: Evidence from Multi-Omics and Single-Cell Atlases

The cross-compartment discordance observed in Section 4 is driven by specific biological mechanisms. High-resolution omics technologies—particularly single-cell transcriptomics and longitudinal proteomics—provide molecular evidence explaining this lung–blood uncoupling.

### 5.1. Omics Mapping of Barrier Permeability

The selective permeability of the alveolar–capillary barrier directly shapes the omics profiles observed in peripheral blood [59]. Longitudinal paired BALF–plasma proteomic analysis in ARDS patients by Mitsuyama et al. corroborated this: the BALF and plasma proteomes overlapped only marginally, consistent with limited macromolecular transit between compartments [60]. Omics data indicate that unless severe diffuse alveolar damage causes significant barrier breakdown, macromolecular immune complexes (such as the NETosis components identified by Lin et al.) are physically retained in the airway. Consequently, the detectable mapping of small-molecule metabolites and specific low-molecular-weight proteins (e.g., ISG15) in plasma omics is consistent with preferential barrier transit; cross-compartment correlation, however, does not by itself establish molecular transport.

### 5.2. Local Immune Autonomy Revealed by scRNA-Seq

Another reason peripheral blood multi-omics fails to fully reflect pulmonary status is the transcriptional autonomy of airway immune cells [61,62]. Seminal single-cell atlases in COVID-19 illustrate this principle. Paired single-cell RNA sequencing (scRNA-seq) atlases demonstrated that pro-fibrotic macrophages and exhausted tissue-resident T cells—which orchestrate local pulmonary infection—are rarely detected in peripheral blood [13,14,63,64]. This implies that blood-based transcriptomics suffers from a “cell-type blind spot,” failing to capture the effector cells actively engaged at the mucosal frontline.

### 5.3. Temporal Asynchrony in Longitudinal Omics

The uncoupling of airway and blood omics signatures is further confounded by the temporal dimension [65]. Local mucosal transcriptional and metabolic remodeling typically initiates within hours of infection, whereas systemic acute-phase omics signatures (such as plasma lipid depletion and the surge of hepatically derived proteins) exhibit a lag. Most current paired studies in non-COVID LRTI employ single-timepoint, cross-sectional designs; the longitudinal exceptions to date are Zhao J et al. [31] (paired sampling at days 1 and 7) and Semenova et al. [30]. In single-timepoint designs, the lung’s early inflammatory phase is aligned with the blood’s delayed systemic response when cross-compartment correlations are computed, so the apparent discordance may partly reflect this temporal mismatch rather than true biological divergence.

### 5.4. Computational Challenges in Cross-Compartment Integration

At the bioinformatics level, the dilution effect of BALF introduces computational challenges for cross-compartment multi-omics integration. Because instilled saline dilutes the alveolar lining fluid, and high-abundance plasma proteins (e.g., albumin) exceed lung-derived markers by several orders of magnitude, signal suppression occurs in non-targeted mass spectrometry [66]. Consequently, direct absolute quantitative comparisons between BALF and plasma omics data introduce methodological noise, which serves as a technical driver of the low inter-compartment Pearson correlation coefficients.

## 6. The Critical Gap and Research Priorities

### 6.1. Why Are Adult Paired Data Lacking?

The paucity of paired airway–blood multi-omics data in adult CAP stems primarily from clinical and ethical constraints. For non-severe adult LRTI, bronchoscopy lacks clinical indications, presenting ethical barriers to BALF acquisition [36]. Even among critically ill patients receiving invasive mechanical ventilation in the ICU, BALF sampling is prioritized for routine microbiological diagnostics, often leaving insufficient residual volumes for multi-omics profiling. Furthermore, adult LRTI multi-omics research has not received the dedicated funding seen during the COVID-19 pandemic.

### 6.2. Why Adult Data Matter

Because current cross-compartment evidence derives from pediatric cohorts or HAP models, it is necessary to question whether these findings can be extrapolated to adult CAP. From an immunological perspective, fundamental differences exist in immune maturity between children and adults; children possess an active thymus and a high proportion of naive T cells, whereas adults accumulate memory T/B cells and exhibit immunosenescence [67]. Second, adult LRTI patients frequently present with comorbidities such as COPD or cardiovascular diseases, which alter baseline metabolic profiles and systemic inflammatory states. Finally, the pathogen spectrum differs (e.g., RSV and *M. pneumoniae* dominate in children, whereas *Streptococcus pneumoniae* prevails in adults) [68]. Therefore, extrapolating pediatric cross-compartment findings to adult LRTI requires dedicated validation.

### 6.3. Four Research Priorities

To overcome the translational bottleneck, future research should prioritize the following four directions, each specified by population, sampling design, sample size, primary outcome, analytical approach, and validation strategy.

(1) Establish adult longitudinal paired cohorts. Proposed population: adult CAP with aetiological stratification (viral vs. bacterial) [69,70], leveraging clinically indicated bronchoscopy in the ICU. Sampling: paired BALF–plasma at admission (days 1, 3, and 7) and at convalescence, with serial paired sampling in at least 20% of participants. Sample size: a target of at least 50 patients. Primary outcome: patient-level concordance of differentially expressed proteins and metabolites between compartments. Analysis: same-platform parallel profiling with per-individual correlation and mixed models. Validation: an independent cohort. The longitudinal design of Semenova et al. (up to five time points over 60 days) provides a template [30].
(2) Evaluate non-invasive airway surrogate sampling strategies. Population: mild CAP in adults and children, for whom BALF is not clinically indicated. Sampling: induced sputum or exhaled breath condensate (EBC) alongside blood [71], with a BALF reference subset of at least 20 participants. Time points: admission and recovery. Primary outcome: concordance of non-invasive airway signatures with BALF-derived signatures, including the “BALF–blood–EBC” metabolite gradient. Analysis: paired concordance analysis. Validation: an independent cohort.
(3) Advance multi-omics integration algorithms. Population: any LRTI cohort with paired airway–blood specimens. Sampling: at least two omics layers per compartment and at least two time points. Sample size: powered for expected multi-layer missingness. Primary outcome: cross-layer shared features and discriminative performance. Analysis: pre-specified supervised latent-variable integration (e.g., DIABLO or MOFA) with permutation testing [72]. Validation: held-out sets and pre-specified reporting standards (data and code availability).
(4) Promote multi-modal data fusion. Population: prospectively registered LRTI cohorts. Sampling: multi-omics features fused with non-omics clinical metadata (pathogen identification, quantitative radiomics, physiological scores) [73,74]. Sample size: at least ten events per candidate predictor. Primary outcome: clinical endpoints (e.g., 28-day outcomes). Analysis: a pre-specified fusion architecture. Validation: prospective external validation and registration.

## 7. Strengths and Limitations

The primary strength of this review lies in its focus on “cross-compartment discordance,” systematically synthesizing the multi-omics literature in non-COVID LRTI. It identifies the void of paired data in adult CAP and discusses the biological mechanisms driving compartmentalization using evidence from single-cell sequencing and longitudinal omics.

However, several limitations must be acknowledged. As a narrative review, it is susceptible to literature selection bias, and the restriction to English-language publications may have omitted relevant studies. The heterogeneity among the included studies regarding omics platforms, specimen processing, and severity grading precluded a quantitative meta-analysis. The small number of included paired studies (*n* = 7) and of those reporting actual cross-compartment comparisons (*n* = 6) objectively reflects the scarcity of data in this field.

## 8. Conclusions

Host functional multi-omics in non-COVID LRTI remains in an early stage of development. Although peripheral blood multi-omics demonstrates potential for pathogen discrimination and severity prediction, paired specimen analyses reveal biological compartmentalization: among the six studies reporting cross-compartment comparisons, the two that reported airway-versus-blood protein inventories found substantially more differentially expressed proteins in the airway than in blood (airway-to-blood DEP count ratios of approximately 3.6 to 9.3); cross-compartment protein correlations were predominantly weak in the one cohort that reported them; and only a subset of molecules, such as ISG15, served as bridging exceptions. Current evidence therefore suggests that peripheral blood provides an incomplete and context-dependent representation of airway-local molecular responses. Bridging this gap through longitudinal paired cohorts, non-invasive airway sampling, advanced integration algorithms, and multimodal data fusion will be essential for translating blood-based multi-omics into clinically useful diagnostics.

## Figures and Tables

**Figure 1:**
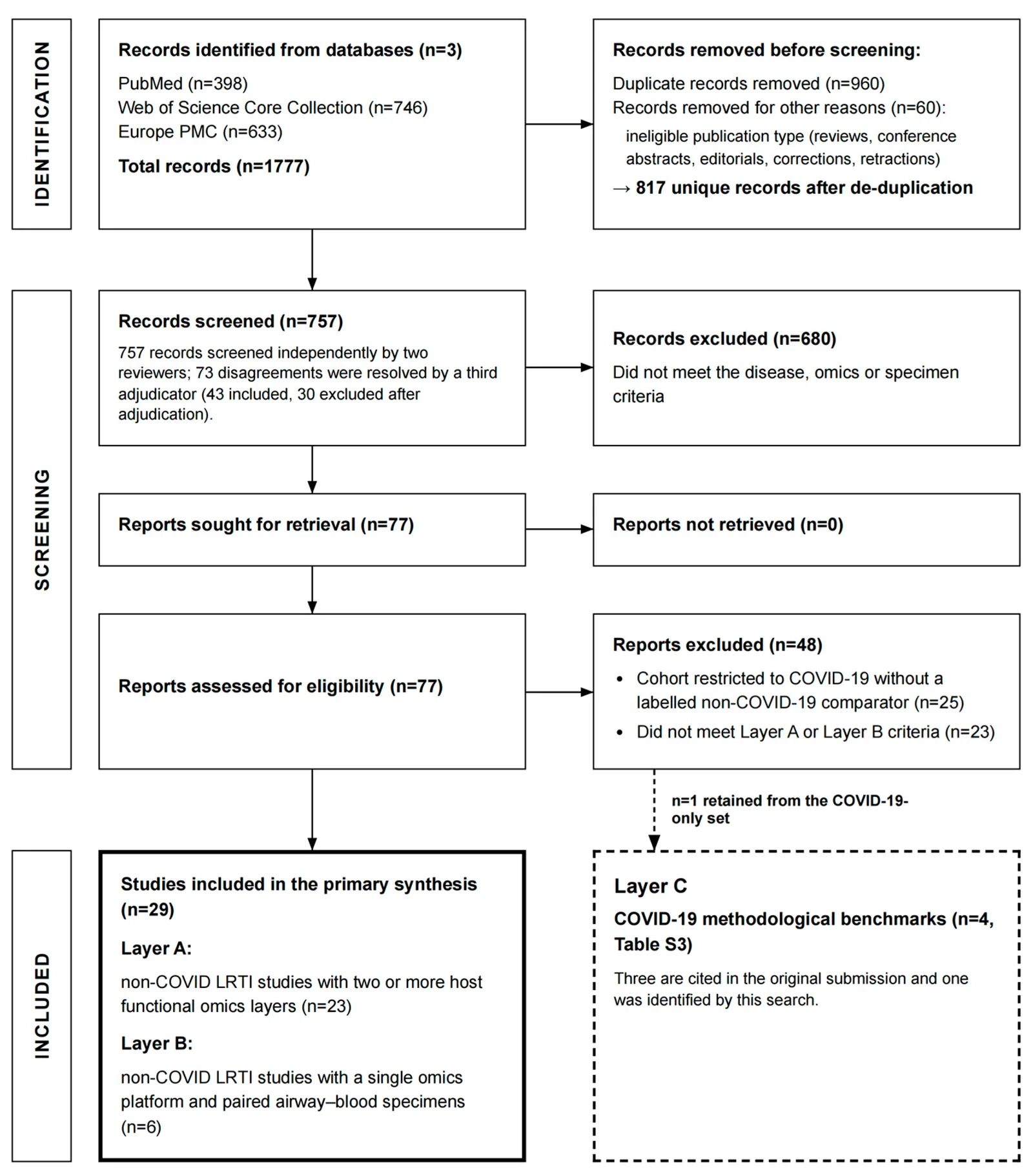
PRISMA 2020 flow diagram of study selection. Records were retrieved on 28 September 2026 from three databases (PubMed, Web of Science Core Collection, and Europe PMC; strategies in Table S1). After de-duplication and removal of ineligible publication types, 757 records were screened independently by two reviewers (73 disagreements resolved by a third adjudicator), and 77 reports were assessed in full text. Forty-eight reports were excluded: 25 COVID-19-only cohorts without a labelled non-COVID-19 comparator (a judgement made at the title/abstract level), and 23 that did not meet the Layer A or Layer B criteria. Twenty-nine studies entered the primary synthesis (Layer A, *n* = 23; Layer B, *n* = 6). The four Layer C COVID-19 methodological benchmarks (Table S3) are listed separately; one of them (Eddins et al. [17]) was identified by the present search. The dashed frame denotes the Layer C COVID-19 methodological benchmarks, and the dashed arrow denotes the one benchmark retained from the COVID-19-only records.

**Figure 2:**
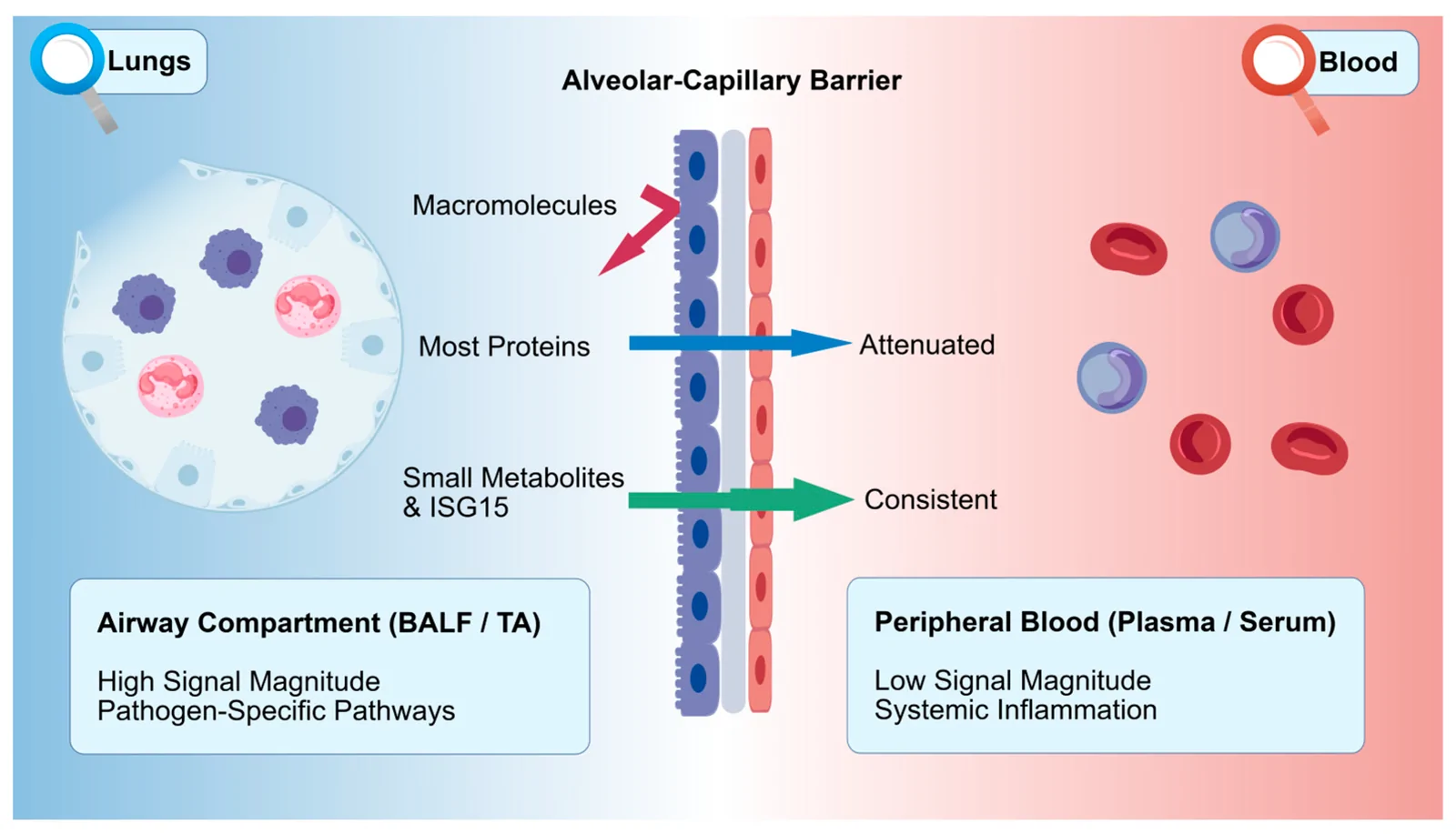
Schematic overview of cross-compartment discordance and its biological determinants in LRTI. (**Left**) The airway compartment captures robust, pathogen-specific molecular signatures with high signal magnitude (e.g., 200 airway versus 56 plasma differentially expressed proteins in viral LRTI [26]). (**Middle**) The alveolar–capillary barrier is proposed to selectively restrict the transit of macromolecular immune complexes (e.g., NETosis components), while small metabolites and ISG15 show consistent signals across compartments; the signals of most proteins are attenuated in blood. (**Right**) Consequently, the peripheral blood compartment shows low signal magnitude dominated by non-specific systemic inflammation, with weak inter-compartment correlations. The critical blind spot: no study has yet applied two or more host omics layers to paired airway–blood specimens from adult community-acquired LRTI (Section 4.4). Blue and red backgrounds indicate the airway and blood compartments, respectively; the blue arrow denotes attenuated protein transit, the green arrow denotes consistent small-metabolite/ISG15 transit, and the dark red arrow indicates restricted macromolecular transit; arrow thickness schematically indicates signal magnitude. Created with BioGDP.com.

**Table 1:** Characteristics of the core included studies. Layer A = non-COVID LRTI studies applying ≥2 host functional omics layers; Layer B = single-omics-platform studies providing paired airway–blood specimens; Table S2 lists all 29 included studies; Layer C benchmarks are listed separately in Table S3. ^a^ Group-level DEP ratios only, no per-individual correlation. ^b^ Five fluids from the same 19 patients, but with each fluid analysed separately.

Layer	Study (Year) [Ref]	Population/LRTI Subtype	N (Analysed)	Specimens (Airway; Blood)	Omics Layers (*n*)	Paired Airway–Blood	Key Findings (Abridged)	Key Limitations (Abridged)
A	Rischke (2025) [18]	Adult CAP, viral vs. bacterial	58 analysed (36 non-COVID LRTI + 22 COVID-19; matched from 69 enrolled)	Plasma; no airway	3	No	EpOME/DiHOME and LAG-3/TRAIL ↑ in viral CAP; CLEC4D/EN-RAGE ↑ in bacterial CAP	Propensity-matched cohort; no external validation; plasma only
A	Wang (2023) [19]	Paediatric severe CAP	127 analysed (73 S-CAP + 54 NS-CAP); 45 healthy controls	Serum; no airway	2	No	5-marker serum panel (CRP, LBP + 3 metabolites) discriminated severity (AUC 0.991)	AUC from discovery cohort; validation by ELISA only; serum only
A	Saballs (2024) [20]	Adult CAP vs. COVID-19 pneumonia vs. controls	146 analysed (71 CAP + 75 COVID-19); 75 controls	Plasma; no airway	2	No	Hypolipidaemia in both pneumonias, more pronounced in COVID-19	Control group from a lipid clinic; COVID-19 comparator; NMR only
A	Tang (2025) [21]	Paediatric *M. pneumoniae* pneumonia	89 analysed (52 + 37 MPP across two cohorts)	Plasma; no airway	2	No	IDO1 ↑ along severity; tryptophan ↓, kynurenine ↑ (Kyn/Trp imbalance)	Cross-sectional; observational; plasma only
A	Kuang (2025) [22]	Adult CAP (severe vs non-severe) vs. controls	46 analysed (46 CAP + 19 controls)	Plasma; no airway	2	No	S1P/APOC1/APOA2 ↓ and S100P ↑ in severe CAP; three-class macro-AUC 0.944	7:3 train/test split, no external validation; single time point
A	Li J (2024) [23]	Adult severe CAP (paediatric comparison set)	74 analysed (31 S-CAP + 43 NS-CAP; 42 disease + 45 healthy controls)	Plasma; no airway	2	No	SAA1/SAA2/CRP ↑, immunoglobulins ↓; lipid metabolism ↓, glycolysis ↑	Single-centre; paediatric comparison retrospective; blood only
A	Lin (2025) [24]	Adult *P. aeruginosa* HAP vs. controls	40 analysed (30 discovery + 10 validation; 30 controls)	BALF + serum (paired)	2	Yes ^a^	93 BALF DEPs vs. 10 key serum DEPs; LCN2/CALR high in airway, attenuated in serum	Group-level DEP ratios only; HAP, not community-acquired LRTI
A	Song (2025) [25]	Paediatric influenza PARDS (pilot)	20 analysed (lung tissue *n* = 2; plasma 20 + 13; BALF *n* = 4)	Lung tissue, BALF; plasma	2	No	KRT17 ↑ in survivors; BALF macrophage shift to resident-like at recovery	Pilot (1 survivor/1 fatality); single time points per compartment
B	Lydon (2025) [26]	Paediatric viral LRTI vs. controls	40 analysed (62 children; 48 paired)	Tracheal aspirate + plasma (paired)	1	Yes	200 TA vs. 56 plasma DEPs; weak TA–plasma correlation except ISG15 (r = 0.79)	Small sample; SomaScan 1.3k panel
B	Chen (2025) [27]	Aspiration vs. non-aspiration pneumonia	19 analysed (10 aspiration + 9 non-aspiration)	BALF, serum, saliva, sputum, urine	1	Yes ^b^	Consistent metabolite trends across fluids; BALF DHEAS correlated with APACHE II (r = −0.619)	*n* = 19; each fluid analysed separately (no compartment discordance test)
B	Zhao (2026) [28]	Severe pneumonia vs. donors	12 analysed (12 patients; 5 donors)	Lung tissue, BALF, blood (matched)	1	Yes	Lung-to-airway sharing of tissue-resident memory-like CD8 T cells via CXCL10–CXCR3	*n* = 12; no BALF from controls; descriptive
B	Wang X (2026) [29]	ARDS (KL-6-assisted subtyping)	137 analysed (166 participants)	Serum + BALF (paired)	1	Yes	3-metabolite panel discriminated severe subtype (AUC 0.917); LPCAT1 candidate target	Small severe paired subgroup; single platform
B	Semenova (2026) [30]	Critically ill adults with pneumonia (Chicago)	26 analysed (7 controls; 6 bacterial; 7 influenza; 13 COVID-19)	BALF + plasma (paired, longitudinal)	1	Yes	Pneumonia type-specific local signatures; antibodies produced locally in the lung	Small subgroups; ICU/ventilated population; single platform
B	Zhao J (2025) [31]	Pulmonary sepsis ARDS ± hypercapnia	61 analysed (50 ARDS + 11 non-sepsis controls)	PBMC + BALF (day 1, day 7)	1	Yes	IL-12p40/IL-23 ↑ D1→D7 in circulation and airways in hypercapnia patients	Monocentric; single platform; ICU ARDS/sepsis population

## Data Availability

No new data were created or analyzed in this study. Data sharing is not applicable to this article.

## Supplementary Materials

The following supporting information can be downloaded at: https://www.mdpi.com/article/10.3390/diagnostics16213489/s1. Table S1: Complete Search Strategies for PubMed, Web of Science Core Collection, and Europe PMC; Table S2: Characteristics of the 29 Included Studies; Table S3: Layer C COVID-19 Methodological Benchmarks. All references cited in the Supplementary Materials (Tables S2 and S3) are included in the main reference list.

## Abbreviations

The following abbreviations are used in this manuscript:

ARDS	Acute respiratory distress syndrome
BALF	Bronchoalveolar lavage fluid
CAP	Community-acquired pneumonia
DEPs	Differentially expressed proteins
EBC	Exhaled breath condensate
HAP	Hospital-acquired pneumonia
ISG15	Interferon-stimulated gene 15
LRTI	Lower respiratory tract infection
mNGS	Metagenomic next-generation sequencing
PBMC	Peripheral blood mononuclear cell
scRNA-seq	Single-cell RNA sequencing
TA	Tracheal aspirate
