# Supplementary material for: Compartmentalization of Host Multi-Omics Responses in Lower Respiratory Tract Infection: Discordance Between Airway-Local and Systemic Molecular Profiles—A Narrative Review
Source: Diagnostics (Basel). 2026 Oct 25;16(21):3489. doi: 10.3390/diagnostics16213489 (PMC13761603; doi:10.3390/diagnostics16213489)
Supplement: Supplementary file 1 [file diagnostics-16-03489-s001.zip › Table S1.pdf]

Table S1. Complete Search Strategies for PubMed, Web of Science Core Collection, and Europe PMC.

Compartmentalization of Host Multi-Omics Responses in Lower Respiratory Tract Infection: Discordance Between Airway-Local and Systemic Molecular Profiles—A Narrative Review

Concept block	PubMed	Web of Science Core Collection (SCI-EXPANDED)	Europe PMC
**Field tag**	[tiab] = Title/Abstract	TS= = Topic (Title + Abstract + Author Keywords + Keywords Plus)	TITLE_ABS: = Title/Abstract
**#1 Disease scope**	"community-acquired pneumonia"[tiab] OR "community acquired pneumonia"[tiab] OR "lower respiratory tract infection"[tiab] OR "respiratory tract infection"[tiab] OR LRTI[tiab] OR pneumonia[tiab] OR ARDS[tiab] OR "acute respiratory distress syndrome"[tiab]	("community-acquired pneumonia" OR "community acquired pneumonia" OR "lower respiratory tract infection" OR "respiratory tract infection" OR LRTI OR pneumonia OR ARDS OR "acute respiratory distress syndrome")	TITLE_ABS:"community-acquired pneumonia" OR TITLE_ABS:"community acquired pneumonia" OR TITLE_ABS:"lower respiratory tract infection" OR TITLE_ABS:"respiratory tract infection" OR TITLE_ABS:LRTI OR TITLE_ABS:pneumonia OR TITLE_ABS:ARDS OR TITLE_ABS:"acute respiratory distress syndrome"
**#2 Omics technologies**	"multi-omics"[tiab] OR multiomics[tiab] OR multiomic*[tiab] OR proteom*[tiab] OR metabolom*[tiab] OR lipidom*[tiab] OR transcriptom*[tiab] OR "single-cell"[tiab] OR "scRNA-seq"[tiab]	("multi-omics" OR multiomics OR multiomic* OR proteom* OR metabolom* OR lipidom* OR transcriptom* OR "single-cell" OR "scRNA-seq")	TITLE_ABS:"multi-omics" OR TITLE_ABS:multiomics OR TITLE_ABS:multiomic* OR TITLE_ABS:proteom* OR TITLE_ABS:metabolom* OR TITLE_ABS:lipidom* OR TITLE_ABS:transcriptom* OR TITLE_ABS:"single-cell" OR TITLE_ABS:"scRNA-seq"
**#3 Specimen types**	"bronchoalveolar lavage"[tiab] OR "broncho-alveolar lavage"[tiab] OR BALF[tiab] OR "tracheal aspirate"[tiab] OR "endotracheal aspirate"[tiab] OR "exhaled breath condensate"[tiab] OR plasma[tiab] OR serum[tiab] OR blood[tiab] OR PBMC[tiab]	("bronchoalveolar lavage" OR "broncho-alveolar lavage" OR BALF OR "tracheal aspirate" OR "endotracheal aspirate" OR "exhaled breath condensate" OR plasma OR serum OR blood OR PBMC)	TITLE_ABS:"bronchoalveolar lavage" OR TITLE_ABS:"broncho-alveolar lavage" OR TITLE_ABS:BALF OR TITLE_ABS:"tracheal aspirate" OR TITLE_ABS:"endotracheal aspirate" OR TITLE_ABS:"exhaled breath condensate" OR TITLE_ABS:plasma OR TITLE_ABS:serum OR TITLE_ABS:blood OR TITLE_ABS:PBMC
**#4 Final combination**	#1 AND #2 AND #3 AND English[lang] AND ("2021/01/01"[PDAT] : "2026/08/31"[PDAT]) AND humans[Filter]	#1 AND #2 AND #3 AND PY=(2021-2026) AND LA=(English)	#1 AND #2 AND #3 AND (PUB_YEAR:[2021 TO 2026]) AND (LANG:"eng")
**Records retrieved (28 September 2026)**	**398**	**746**	**633**

Notes

a. Equivalent concept blocks across databases. The three databases were searched with identical concept blocks (disease scope, omics technologies, specimen types), combined with the Boolean operator AND. Only the field-tag syntax differs between platforms; no concept was added or removed for any database so that the three searches are directly comparable.

b. Field scope. PubMed [tiab] and Europe PMC TITLE_ABS: search the title and abstract fields only. Web of Science TS= (Topic) searches the title, abstract, author keywords, and Keywords Plus. Keywords Plus terms are generated automatically from the titles of a record's cited references and are indexed as free text by Clarivate; this makes TS= the broadest of the three field scopes and contributes to the higher record count retrieved from Web of Science.

c. No controlled vocabulary was applied. MeSH (PubMed) and Emtree (Embase) terms were deliberately not used. Exploding MeSH subtrees inflates recall by retrieving records whose indexing reflects any descendant term, irrespective of the study's actual content, and there is no controlled vocabulary in Web of Science that can be applied equivalently. Restricting all three searches to free-text title/abstract searching keeps the concept blocks strictly parallel and the three retrievals comparable. The cost is reduced sensitivity; this was evaluated by back-testing (see Note g).

d. Truncation and phrase searching. An asterisk (*) truncates a term to its stem (e.g., proteom* retrieves proteomic, proteomics, proteome, proteomics-based). Multi-word concepts were entered as quoted phrases. Hyphenated and unhyphenated variants of the same concept were both searched (e.g., "community-acquired pneumonia" OR "community acquired pneumonia"; "bronchoalveolar lavage" OR "broncho-alveolar lavage") because hyphenation is not normalised consistently across the three indexing systems.

e. Limits. All three searches were limited to English-language publications and to 2021 onward. PubMed additionally applied the humans[Filter] limit; the equivalent organism limit was not applied in Web of Science or Europe PMC because neither platform offers a directly equivalent filter and applying it to only one database would have broken the parallelism of the concept blocks. PubMed and Europe PMC applied a specific end date (2026/08/31); the Web of Science PY= and Europe PMC PUB_YEAR filters operate at year-level granularity and therefore retrieve the whole of 2026. Three Web of Science records carrying a publication date after 31 August 2026 (10, 19 and 21 September 2026) were retrieved on this basis. They were screened identically to all other records and none met the inclusion criteria.

f. Deduplication. Records retrieved from the three databases were merged and de-duplicated by exact normalised title matching, with DOI and PubMed identifier used as secondary merge keys only where title similarity was at least 0.5 (Jaccard index on title tokens). This yielded 817 unique records from 1777 database records; 960 duplicates were removed.

g. Sensitivity back-testing, and relation to the strategy of the original submission. The revised strategy was back-tested against nine studies already known to the authors to be relevant (the six studies that formed the core multi-omics evidence base of the original submission, together with three paired airway–blood studies). All nine were retrieved (9/9) by the three-database searches reported above. The PubMed-only strategy used in the original submission did not achieve this: re-running it retrieved 175 records, from which three of the nine back-test studies were not retrievable. Per-block testing localised three independent causes: (i) the omics block listed only plural forms with no truncation, so records indexed under the singular form were missed (e.g. Rischke et al., “Proteomic, metabolomic and lipidomic profiles…”); (ii) the specimen block did not include blood, so records indexed only under that term were missed (e.g. Saballs et al., which reports “blood lipidomes”); and (iii) the PubMed search applied the humans[Filter] limit, which excludes records held in PubMed but not indexed in MEDLINE (e.g. Kuang et al.). The revised three-database strategy corrects all three: omics terms are truncated (proteom*, metabolom*, lipidom*, transcriptom*), blood is included in the specimen block, and the non-MEDLINE records are retrieved through Web of Science and Europe PMC. All numbers in the revised Figure 1 and Table 1 derive from this single search.

h. Search date. All three searches were executed on 28 September 2026. Record counts are those returned on that date and are not reproducible at a later date because all three databases are continually updated.

Records identified, by database

Database	Records identified	Records unique to this database
PubMed	398	6
Web of Science Core Collection (SCI-EXPANDED)	746	156
Europe PMC	633	45
**Total (before de-duplication)**	**1777**	**—**

After de-duplication, 817 unique records remained and were screened against the eligibility criteria (Figure 1). Records were shared with each other by the three databases as follows: retrieved by all three databases, n = 347; Europe PMC and Web of Science, n = 218; Web of Science only, n = 156; Europe PMC only, n = 45; PubMed and Web of Science, n = 24; PubMed and Europe PMC, n = 21; PubMed only, n = 6.
